# Golf‐Related Injuries in Adolescent Golfers: A Scoping Review

**DOI:** 10.1002/hsr2.71818

**Published:** 2026-03-10

**Authors:** Stephen Lee, Michele Lastella, Andrew Vitiello, Henry Pollard

**Affiliations:** ^1^ School of Health, Medical, and Applied Sciences Central Queensland University Perth Western Australia Australia; ^2^ Appleton Institute for Behavioural Science Central Queensland University Adelaide South Australia Australia; ^3^ Private Practice Sydney New South Wales Australia; ^4^ Central Queensland University Rockhampton Queensland Australia; ^5^ Faculty of Health Sciences Durban University of Technology Durban KwaZulu‐Natal South Africa

**Keywords:** adolescent, child, golf, injury, young adult

## Abstract

**Objectives:**

This scoping review aims to systematically explore the existing literature on golf‐related injuries in adolescent golfers aged 10–19 years, focusing on injury prevalence, management strategies, and the relationship between training volume and injury risk.

**Design:**

A scoping review methodology was employed, adhering to PRISMA extension guidelines. A comprehensive search across multiple databases was conducted to identify relevant studies on injuries sustained by adolescent golfers.

**Method:**

A systematic search was performed across nine databases, including CINAHL, COCHRANE, and PUBMED, utilizing keywords related to adolescence, injury, and golf. Articles were included if they reported on golf‐related injuries among participants aged 10–19 years. After screening, 14 studies met the inclusion criteria.

**Results:**

Findings reveal a significant gap in the literature regarding injury profiles of adolescent golfers, with most studies conflating data with adult populations. The majority of studies addressing golf‐related injuries in adolescents focus on injuries not directly associated with the act of playing golf. Specific epidemiological data and tailored injury management strategies remain sparse. Additionally, no literature currently correlates training volume with injury risk in this demographic.

**Conclusions:**

This review highlights the need for targeted research on the unique injury risks faced by adolescent golfers. Existing studies predominantly address injuries related to external factors rather than those arising from playing golf itself. Future investigations should prioritize delineating injury profiles of adolescent golfers to inform effective injury management and prevention strategies, ultimately enhancing safety and performance in this growing demographic.

## 1 Introduction

Golf is one of the most widely participated in sports in Australia, with approximately 1.5 million individuals engaging in the sport on the golf course (accounting for 6.4% of the population) and an additional 300,000 individuals opting for golf simulation experiences (representing 1.2% of the population) [1]. Participation in junior golf, defined as individuals under 18 years of age, has experienced a significant increase, with growth rates of 2%–6% between the years of 2019 and 2022 [1, 2, 3]. The Golf Australia MyGolf junior program, catering to children aged 5–12, demonstrated a 13% increase in participation during the 2021/2022 period, while overall junior participation under 18 years increased by 4.2% in the same timeframe [1]. While this accelerated participation growth was likely amplified by the COVID‐19 pandemic—during which golf's outdoor nature and physical distancing compatibility increased its appeal—the trend towards youth involvement in golf was already evident and may be linked to a growing aspiration among adolescent golfers to pursue professional careers, particularly in light of the lucrative opportunities presented by the American and European golf tours [4]. The proportion of PGA Tour victories by players aged 25 years or younger has increased from 10% between 1987 and 2016 to nearly 25% from 2016 to 2020 [4], with a three‐fold increase in PGA Tour wins by players aged 23 years or younger since 2012 compared to the preceding decade [4]. The financial ramifications of such achievements are substantial; in 2020, four players under the age of 25 ranked among the top 25 PGA Tour Money Leaders, with individual earnings ranging from $2.8 million (USD) to $5.2 million (USD) [5]. In 2022, the establishment of LIV Golf, a new golf league, further underscored this trend by offering individual tournament winners $4 million (USD), with even the last‐place finisher guaranteed $120,000 (USD) [6].

Increased interest in golf amongst adolescents has created a drive towards elite adolescent golf programs such as the Golf Australia High Performance Program, Golf New South Wales High Performance Program, and Golf Western Australia High Performance Program, whose aim is to support elite golfers onto the professional circuit [7]. This trend has resulted in these young athletes engaging in high‐intensity training and sport specialization at increasingly younger ages, which is often accompanied by heightened performance pressures [8]. These factors all play a role in acute and chronic overuse injury risk, burnout, and dropping out of sport(s) [9]. Research indicates that athletes who specialize early are at an elevated risk for injuries [10]. Consequently, overuse injuries that were once predominantly seen in older athletes are now becoming more common among adolescents [10, 11]. Specific injuries, such as patellofemoral pain syndrome, have been observed with increasing frequency in specialized youth athletes participating in sports like basketball, soccer, and volleyball [12].

Many studies have been published describing the injury profile of amateur and elite adult golfers [13, 14], but most studies assessing injuries in golfers do not include adolescents [15, 16, 17, 18, 19], a demographic defined by the World Health Organization as individuals aged between 10 and 19 years [20]. Unfortunately, no data currently exists on the epidemiology of golf‐related injury in adolescents. Unlike adults, adolescents face distinct injury risk factors, including growth spurts, maturity‐associated variations, and a lack of complex motor skills required for certain sports [8, 21]. Given that the majority of existing literature on golf injuries has concentrated on adult populations, this scoping review aims to systematically explore the available literature concerning the injury profiles of elite adolescent golfers, focusing on epidemiological data, current injury management strategies, and training volumes.

## 2 Methods

This scoping review includes the essential reporting items as outlined in the PRIMSA extension for scoping reviews.

### 2.1 Stage 1: Identifying the Research Question

This review aimed to identify the epidemiology of golf injuries, the current injury management strategies, and determine if training volume is correlated with injuries in adolescent golfers. The specific aims were:

### 2.2 Identify Epidemiological Data on Golf Injuries in Adolescent Golfers Between 10 and 19 Years of Age

- Determine the type, location, frequency, and management strategies of injuries occurring in adolescent golfers
- Determine if training volume is related to injury
- Determine current injury management and prevention strategies used by adolescent golfers

### 2.3 Stage 2: Identifying Relevant Studies

As this was a scoping review, the search included a wide range of databases, including CINAHL, COCHRANE, EBSCOHOST, EMBASE, EMCARE, Index to Chiropractic Literature, PUBMED, SCOPUS, and Web of Science. Database searches were performed in November 2025. The following keywords and MeSH terms were used: adolescence, adolescent, child, juvenile, teen, teenager, youth, young adult, injury, and golf. The search strategy was reviewed by a senior academic librarian. The final search strategy for CINAHL can be found in Table 1. The final search results were exported into a spreadsheet, and duplicates were removed by the lead researcher, SL.

**Table 1:** CINAHL search strategy (literature search performed 9 November 2025).

	Search terms and combinations	Search results	Limits applied
S1	(MH “Adolescence + “) OR (MH “Child + “)	1,200,528	Expanders—Apply equivalent subjects Search modes—Find all my search terms
S2	“injury” OR (MH “Athletic Injuries + “)	266,374	Expanders—Apply equivalent subjects Search modes—Find all my search terms
S3	“golf” OR (MH “Golf Injuries”)	1,930	Expanders—Apply equivalent subjects Search modes—Find all my search terms
S4	(S1 AND S2 AND S3)	60	Expanders—Apply equivalent subjects Search modes—Find all my search terms

### 2.4 Stage 3: Study Selection

To be included in this scoping review, articles needed to include participants between the ages of 10–19 years. They also needed to study or report on injuries related to the action of playing golf. The lead researcher assessed the titles and abstracts from the search results, which produced 18 articles. The full‐text articles were then retrieved and screened, which resulted in the exclusion of four articles as they did not include adolescents or were not describing an injury from playing golf, such as trauma from a golf cart or getting hit by a golf ball. This left a remainder of 14 articles to be included.

### 2.5 Stage 4: Data Extraction

Data was extracted to a pre‐formatted Microsoft Excel spreadsheet: author, year, title, sample size, and injury mechanism. The data was copied and pasted across where applicable to avoid potential errors. Injury mechanism was defined by the underlying causal factor of the reported injury. This included trauma from a golf cart, being hit by a golf ball or club, environmental factors such as heat stroke or lightning, or injury from the actions of playing golf. It is important to define injury mechanism in this scoping review as the literature on adolescent golf‐related injuries can occur from a variety of sources. The main distinguishing feature was whether the reported injury occurred from an action related to playing golf or from other external factors.

### 2.6 Stage 5: Collating, Summarising and Reporting the Results

Through this reporting process, we endeavor to identify any existing literature on the epidemiology of golf injuries, current injury management strategies, and whether training volume is correlated with injuries in adolescent golfers. The studies included in this scoping review are summarized in Table 2.

**Table 2:** Studies included in this scoping review.

Author, year	Title	Study design	Sample size	Key findings
Batt, M. E., 1992	A survey of golf injuries in amateur golfers	Survey	193	57% reported injuries Wrist, back, muscle, elbow and knee problems were most likely to compromise a player's game. The occurrence of injury among men (56%) was marginally lower than in women (59%). Back injuries particularly affected men, and elbow injuries women. Overuse and poor technique were the main etiological factors.
Gosheger et al., 2003	Injuries and overuse syndromes in golf	Retrospective cohort study	703	83% reported injuries involved overuse and 17% were single trauma events. Professional golfers were injured more often, typically in the back, wrist, and shoulder. Amateurs reported many elbow, back, and shoulder injuries. Severity of injuries was 52% minor, 27% moderate, and 22% major. Carrying one's bag proved to be hazardous to the lower back, shoulder, and ankle. Warm‐up routines of 10 min or greater had a positive effect.
Evans, M. W., 2004	Hamate hook fracture in a 17‐year‐old golfer: importance of matching symptoms to clinical evidence	Case report	1	17‐year‐old golfer with persistent left wrist pain for four months that began while playing golf. Approximately one week after reporting the injury, he was diagnosed with a scaphoid fracture.
Fradkin et al., 2005	Golf injuries ‐ Common and potentially avoidable.	Survey	522	35% reported a golfing injury within the previous 12 months. Lower back was the most commonly injured body region. Strains were the most frequent type of injury (68%). Of the 184 injuries reported, 154 sought treatment from a health professional. Physiotherapists were the most common health professional consulted. Performance was affected in 79% of cases, with 70% of the injured golfers missing games or practice sessions due to injury.
Fradkin et al., 2006	Opportunities for prevention of golfing injuries	Retrospective study	547	This study reviewed presentations to an emergency department. Injuries in males outnumbers females 3:1, while the proportions of participants in each age group were similar. Most injuries were sustained by being struck by a ball or club or through a collision with another person. Falls were the other main mechanism of injury. Head injury was the most common reason for presentation at an emergency department, accounting for over one‐third of all cases. Golfers older than 65 years sustained a higher proportion of injuries related to falls and a higher‐than‐expected proportion of lower extremity injuries. Golfers younger than 15 years had more head, neck and face injuries due to being struck by an object. Open wounds were the most common type of injury, followed by strains and sprains.
Faustin CM et al., 2007	Isolated posterior labrum tear in a golfer: a case report	Case report	1	17‐year‐old male, right‐hand‐dominant golfer experienced a “pop” and acute onset of pain in the back of his left non‐dominant shoulder as he began his forward swing with a driver. He sought treatment 14 days later from his primary physician and a course of nonoperative treatment was initiated. Six months later, the pain returned and a magnetic resonance imaging arthrogram found a posterior glenoid labral tear.
Bugbee S, 2010	Rib stress fracture in a golfer	Case report	1	A 15‐year‐old, male, right‐handed golfer, noticed a gradual onset of left mid to upper back pain during a high school golf match. A computerized tomography scan found the left posterior second rib demonstrated a healing fracture directly adjacent to the transverse process of the T2 vertebral body.
Rolison CJ 4th & Smoot MK., 2017	Hand pain in a golfer: a case report of a metacarpal stress injury and a review of the literature regarding return to play in grip athletes	Case report	1	19‐year‐old golfer with a two‐week history of dominant‐hand pain after several months of daily golf. Magnetic resonance imaging illustrated a grade 3 (Fredericson Classification System) stress reaction of the second metacarpal without a fracture line.
Bueno et al., 2018	Injury prevalence across sports: a descriptive analysis on a representative sample of the Danish population	Survey	3498 adults 3221 children	43 adolescents participated in golf in a 12‐month period. One participant (2%) had reported an injury.
Brearley SL et al., 2021	Inter‐disciplinary conservative management of bilateral non‐united lumbar pars defects in a junior elite golfer	Case report	1	A 15‐year‐old elite male golfer reported ongoing (approximately 12 weeks) low back pain of gradual onset. A magnetic resonance imaging scan 6 months later showed pars fractures bilaterally with 1 mm and 2 mm defects both at acute angles.
Quinn et al., 2022	Increased trunk muscle recruitment during the golf swing is linked to developing lower back pain: A prospective longitudinal cohort study	Prospective longitudinal cohort study	33	After a 6‐month monitoring period, 17 participants developed lower back pain. Increased dominant rectus abdominis and dominant latissimus dorsi during the golf swing is linked with developing lower back pain. Training strategies aimed at reducing these muscles activation during the swing may reduce the incidence of lower back pain in young elite male golfers.
Karpudewan J. & Badia A., 2023	Occult lunotriquetral ligament injuries in adolescent golfers	Case study	3	Three case series of highly competitive adolescent golfers who presented with persistent and intractable ulnar‐sided wrist pain. The lunotriquetral ligament injury was confirmed solely via wrist arthroscopy.
Twomey‐Kozak et al., 2024	Estimates of golf‐related upper extremity Injuries in the United States: A 10‐Year Epidemiology study (2011‐2020)	Epidemiology Study	1862	Individuals aged 10 to 19 years were among the most injured age groups. This age group accounted for 18.4% of all golf‐related upper extremity injuries presenting to emergency departments. There were an estimated 14,843 injuries in this age bracket over the 10‐year period.
Hamada et al., 2025	Warm‐up program for adolescent golfers reduces low back pain: a double‐blind, randomized controlled trial	Double‐blind, randomized controlled trial	45	There was no significant difference in the number of people who experienced low back pain between those in the Golfer's Low Back Pain Exercise Prevention program (GLEP) and those in the sham group. The number of LBP incidents was significantly lower in the GLEP group (16 incidents) compared to the sham group (100 incidents), representing an 84% reduction in low back pain incidents.

## 3 Results

### 3.1 Search Results and Study Selection

**Figure 1:**
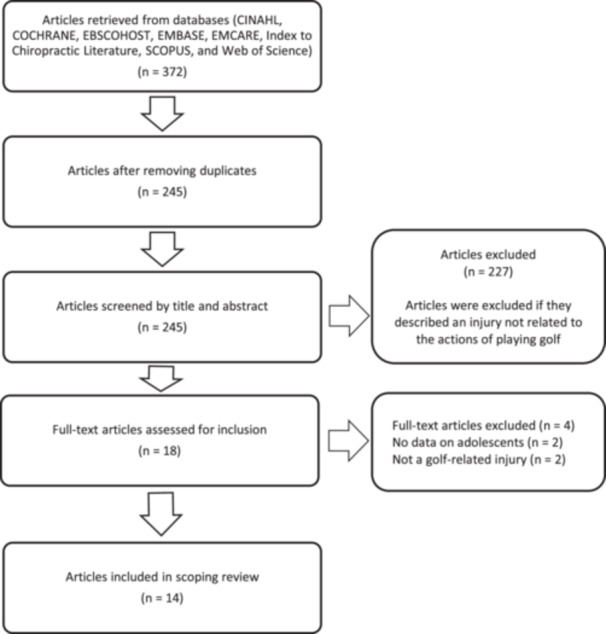
PRISMA flow diagram.

The search was conducted in November 2025 and produced 372 publications. After removing duplicates, 245 articles were screened by title and abstract. Eighteen articles met the inclusion criteria, and the full article was retrieved for further review. Fourteen studies were included in this scoping review. Refer to Figure 1 for the flow diagram from identification through to final inclusion.

### 3.2 Characteristics of Included Studies

The characteristics of included studies investigating injuries in adolescent golfers are reported in Table 2.

### 3.3 Epidemiological Data on Adolescent Golfers

Age was not considered to be a factor in determining injury risk, as Gosheger et al. (2003) found no significant difference in injury prevalence or distribution across different age groups ranging from age 10 to 82 years.

### 3.4 Injury Profile of Adolescent Golfers

Most other research studies have encompassed participants across a wide age range, from 10 to 85 years, without distinguishing results by specific age groups [16, 22, 23, 24]. Consequently, while these studies incorporated adolescents, the data pertaining to this demographic is conflated with that of adults. Nevertheless, Gosheger et al. (2003) reported an injury rate of 2.07 per amateur golfer within the age range of 10–82 years [22]. Batt (1992) indicated a higher injury occurrence of 57% among amateur golfers aged 17–85 years, although this figure includes injuries resulting from being struck by a ball or club, blisters, attacked by bees, or lacerations. The study also found the occurrence in men (56%) was marginally lower than in women (59%) [16]. Back pain emerged as the most prevalent issue, particularly among men (52% of men vs. 29% of women), whereas women were more frequently affected by elbow injuries [16]. In competitive female golfers aged 16–75, an injury incidence rate of 35% was documented [23]. Additionally, Quinn et al. (2022) reported a significant incidence of low back pain, affecting 41% of young male elite golfers aged 16 to 30 [24].

Three studies provided insight specifically on the adolescent group. Fradkin et al. (2006) identified that the highest frequency of golf‐related injuries occurred in individuals aged 10–19 years, who accounted for 20% of the 547 golf‐related injuries recorded in an emergency department over a 5‐year duration [25]. This age group has been further corroborated by a recent 10‐year epidemiology study (2011–2020), which highlighted adolescents aged 10–19 years as one of the most injured populations in golf, accounting for 18.4% of all golf‐related upper extremity injuries presenting to emergency departments in the United States [26]; notably, golfers under the age of 15 also had more head, neck, and face injuries due to being struck by an object [25]. Conversely, Bueno et al. (2018) reported that only one out of 43 golfers aged 7–15 years (2%) suffered an injury over a 12‐month period [27].

### 3.5 Training Volume and Its Relation to Injury

No literature currently exists in measuring the effects of training volume on injuries in adolescent golfers.

### 3.6 Current Injury Management and Prevention Strategies in Adolescent Golfers

A recent investigation into a Golfer's Low Back Pain Exercise Prevention program (GLEP) designed for adolescent golfers revealed that approximately 53.3% of participants experienced low back pain (LBP) within the preceding year [28]. This discomfort was frequently associated with the utilization of drivers and full‐shot clubs, with the onset of pain typically occurring after the execution of 10–30 shots. Although the intervention did not lead to a statistically significant reduction in the prevalence of LBP among participants, it did result in a substantial decrease in the overall incidence of LBP. Specifically, the GLEP cohort reported only 16 incidents of LBP, in contrast to the 100 incidents recorded in the sham group, culminating in an approximate 84% reduction in LBP incidents over a 12‐week period [28].

## 4 Discussion

### 4.1 Injuries in Adolescent Golfers

Limited research exists regarding injuries sustained by adolescent golfers, as the majority of studies investigating golf‐related injuries have not included participants aged 10–19 years [15, 16, 17, 18, 19]. Among the few studies that examined adolescent golfers, the emphasis was placed on injuries that are not directly associated with the act of playing golf. These included conditions such as heat stroke [29], golf cart‐related injuries [30], golf ball‐related eye [31, 32] and craniofacial injuries [33, 34, 35, 36, 37, 38, 39], trauma from being struck by a golf club [14, 40, 41, 42], or presentations at trauma or emergency centers [14, 43, 44]. Individual case reports have documented instances of rib fracture [45], lumbar pars defect [46], posterior labrum tear [47], and hand and wrist injuries [48, 49, 50].

Notably, numerous studies have highlighted cases of accidental head trauma within the adolescent age group, often resulting from impacts with a golf ball or club [14, 33, 34, 35, 36, 37, 38, 39, 40, 41, 42, 51]. While such incidents are relatively infrequent, they are underscored in the literature due to their potentially severe outcomes, including fatalities [51, 52]. Research indicates that boys aged 5–9 years are particularly vulnerable, with the primary causes being unsupervised play with golf clubs, or standing in close proximity to another player hitting the ball [23, 51, 52].

The injury epidemiology pertaining to adolescent golfers has garnered limited scholarly focus within the existing body of literature [22, 25, 27]. A recent investigation involving 43 adolescents who engaged in golf over a 12‐month period revealed that only one participant (2%) reported sustaining an injury [27]. It is crucial to recognize that this finding is part of a broader study aimed at assessing the prevalence of sports injuries within the Danish population, which included a sample of 3498 adults and 3221 children and adolescents. It is important to note that the categorization of participants aged 7–15 as children and adolescents, while those over 15 were classified as adults, may limit the representativeness of this result concerning the adolescent population [27]. Further, Gosheger et al. (2003) found no significant difference in injury prevalence or distribution across varying age groups, concluding that age did not influence injury risk. However, their sample included participants ranging from 10 to 82 years, with only 22 (3.1%) being adolescents, which may not adequately represent this cohort. In fact, the professional golfers in their study were all over the age of 21 years, potentially skewing the results. Although an overall injury rate of 3.06 per professional player and 2.07 per amateur was reported, specific injury rates for the adolescent group were not provided [22].

The only other study focusing on adolescent golfers was conducted by Fradkin et al. (2006), which documented golf‐related injuries presenting at an emergency department over a 5‐year period. Of the 547 recorded presentations, 20% were individuals aged 10–19 years, marking this age group as having the highest incidence of presentations. The authors noted that golfers under 15 years of age experienced a greater number of head, neck, and face injuries due to being struck by an object [25]. However, it is crucial to acknowledge that this study may not represent the entire golfing population, as many injuries do not necessitate emergency care. Consequently, the setting for data collection in this study likely influenced the types and severity of injuries reported.

### 4.2 Injuries in Elite Adolescent Golfers

Two studies have investigated injury rates among competitive and elite golfers spanning a broad age range from 16 years and older. However, these studies did not categorize participants into distinct age groups, which limits the ability to extract injury data specifically for the adolescent cohort [23, 24]. Fradkin et al. (2005) reported an injury incidence rate of 35% among 522 competitive female golfers aged between 16 and 75 years [23]. Similarly, Quinn et al. (2022) documented a LBP incidence of 41% in a limited sample of 41 young male elite golfers aged 16–30 years. It is noteworthy that participants with pre‐existing serious spinal or hip conditions were excluded from this study, which primarily aimed to assess differences in spinal muscle activity on electromyography among those experiencing LBP [24]. Furthermore, both studies concentrated exclusively on one sex, which is a critical factor to consider, given that the swing mechanics of elite female golfers differ significantly from their male counterparts [53].

While existing data on adult golfers' injuries is available, there is a pressing need for dedicated research focused on adolescent golfers. The literature indicates that adolescents undergo structural and tissue changes that may heighten their injury risk [54, 55]. For instance, Prum et al. (2023) discovered that early engagement in golf practice, particularly before the age of 12 years, could lead to alterations in bone morphology [56]. They attributed this to the repetitive torsional stresses on the lead leg during the follow‐through phase of a golf swing, potentially resulting in increased internal rotation of the lead lower limb. Such adaptations may predispose individuals to degenerative knee arthropathy later in life. Consequently, the authors proposed several recommendations for young golfers to reduce the high torsional stress on the lead lower limb, including the adoption of partial swings, a more open follow‐through foot position, reduced swing intensity, and practicing on the non‐dominant side (i.e., reverse swing) to balance stress distribution on the lower limbs [56].

In conclusion, it is evident that a significant gap exists in the literature concerning adolescent golfers. Addressing this gap through targeted research would yield a more comprehensive understanding of injury profiles within this unique population and facilitate the development of effective risk management strategies tailored to adolescent golfers.

### 4.3 Injury Prevention Strategies

The findings from a recent study investigating injury prevention strategies indicate that, although a warm‐up program may not significantly affect the overall prevalence of LBP, it plays a vital role in diminishing the severity and frequency of LBP episodes among adolescent golfers [28]. It is important to acknowledge that this study was limited by a small sample size of only 45 adolescents. The effectiveness of the GLEP was particularly pronounced during specific trunk movements, including trunk extension and rotational actions, which are essential during the golf swing. Notably, the occurrence of LBP was reduced across various phases of the golf swing, with the exception of the “address” phase and instances classified as “unknown” [28]. These findings suggest that targeted warm‐up strategies can enhance biomechanical preparedness and potentially reduce the risk of injury during high‐stress movements. In summary, while the current landscape of injury prevention for adolescent golfers remains underdeveloped, evidence indicates that structured warm‐up programs, such as the GLEP, can contribute to a reduction in the incidence of LBP. Continued research and the formulation of comprehensive injury prevention strategies are crucial for safeguarding the health and performance of adolescent golfers.

## 5 Conclusion

In summary, the current body of literature pertaining to injuries among adolescent golfers inadequately delineates the injury profile specific to this cohort. Most studies that have included this demographic do not differentiate between adolescents and adults, resulting in a limited understanding of their unique injury risks. The majority of research has predominantly concentrated on injuries associated with golf carts, golf balls, or golf clubs, rather than those arising from the act of playing golf itself. To enhance our understanding of this area, it is imperative that future investigations focus specifically on the adolescent population, thereby providing a more precise injury profile. Additionally, it would be advantageous for forthcoming studies to examine injuries linked to golf‐related activities, as this would shed light on the relationship between training volume and injury risk. Such insights could pave the way for more informed recommendations regarding injury management and prevention strategies.

## Author Contributions

**Stephen Lee:** conceptualization, investigation, methodology, project administration, resources, validation, visualization, writing – original draft, writing – review and editing. **Michele Lastella:** supervision, writing – review and editing. **Andrew Vitiello:** supervision, writing – review and editing. **Henry Pollard:** conceptualization, supervision, writing – review and editing.

## Disclosure

All authors have read and approved the final version of the manuscript. The lead author, Stephen Lee, had full access to all of the data in this study and takes complete responsibility for the integrity of the data and the accuracy of the data analysis.

## Conflicts of Interest

The authors declare no conflicts of interest.

## Transparency Statement

The lead author Stephen Lee affirms that this manuscript is an honest, accurate, and transparent account of the study being reported; that no important aspects of the study have been omitted; and that any discrepancies from the study as planned (and, if relevant, registered) have been explained.

## Data Availability

The authors confirm that the data supporting the findings of this study are available within the article. Data sharing not applicable to this article as no datasets were generated or analyzed during the current study.
